# Contrasting reproductive strategies of two Hawaiian *Montipora* corals

**DOI:** 10.1038/s41598-022-16032-6

**Published:** 2022-07-18

**Authors:** E. Michael Henley, Mariko Quinn, Jessica Bouwmeester, Jonathan Daly, Claire Lager, Nikolas Zuchowicz, Daniel W. Bailey, Mary Hagedorn

**Affiliations:** 1 Smithsonian Conservation Biology Institute, Front Royal, VA 22630 USA; 2 Hawaiʻi Institute of Marine Biology, University of Hawaiʻi at Mānoa, 46-007 Lilipuna Rd, Kaneohe, HI 96744 USA

**Keywords:** Marine biology, Tropical ecology

## Abstract

Sessile invertebrates often engage in synchronized spawning events to increase likelihood of fertilization. Although coral reefs are well studied, the reproductive behavior of most species and the relative influence of various environmental cues that drive reproduction are not well understood. We conducted a comparative examination of the reproduction of the well-studied Hawaiian coral *Montipora capitata* and the relatively unknown reproduction of its congener*, Montipora flabellata*. Both are simultaneous hermaphroditic broadcast spawners that release egg-sperm bundles with external fertilization. *Montipora capitata* had a distinct reproductive pattern that resulted in coordinated gamete maturation and the synchronized release of thousands of egg-sperm bundles across two spawning pulses tightly coupled to consecutive new moon phases in June and July. *Montipora flabellata* exhibited a four month reproductive season with spawning that was four-fold less synchronous than *M. capitata*; its spawning was aperiodic with little linkage to moon phase, a broadly distributed release of only dozens or hundreds of bundles over multiple nights, and a spawning period that ranged from late June through September. The reproductive strategy of *M*. *flabellata* might prove detrimental under climate change if increased frequency and severity of bleaching events leave it sparsely populated and local stressors continue to degrade its habitat.

## Introduction

Coral reefs and other marine fauna are facing unparalleled threats to their future integrity due to the dual consequences of ocean acidification and warming from climate change^1–3^, local habitat degradation^4^, and emergent diseases^5,6^. The persistence of these populations in the Anthropocene will hinge upon their ability to survive and successfully reproduce. Although corals can reproduce asexually by fragmentation and also sexually through spawning events^7,8^, their reproduction is being negatively impacted by various sources of coastal pollution^9–11^ and repeated bleaching events fueled by ocean warming from climate change^12–14^. Asexual reproduction generates ramets that are genetically identical (though the accumulation of somatic cell mutations may occur^15^) and can lead to few competitive genotypes occupying large tracts of reef^16^. In contrast, sexual reproduction leads to the production of new genotypes via gene recombination, which enhances genetic diversity within populations and is key to driving adaptation^17^. When sexual reproduction is impaired, the loss of genetic diversity that might help populations adapt to rapidly changing oceans is at risk.

While some scleractinian corals brood and release settlement-competent larvae (~ 10–15% of species), more than 60% of species studied to date are hermaphroditic broadcast spawners that release egg-sperm bundles in the water column^7,8,18,19^. In these species, because fertilization occurs externally and their gametes are short-lived, the successful union of egg and sperm is often considered incumbent upon synchronous spawning of gametes^20,21^. Many coral species around the world have been documented to spawn their gametes in tightly coordinated windows, and synchronized, multi-species spawning events have been reported from Australia, the Red Sea, the Caribbean, Japan, Africa, the greater Indo-Pacific, as well as equatorial regions such as Singapore and Malaysia^7,8,18,22–24^.

Gametogenesis and the synchronized release of gametes are typically influenced by external stimuli, including long-term seasonal temperature and insolation changes, short-term lunar cycles, and immediate cues of time after sunset or before sunrise^18,25–28^. Other environmental cues such as tide cycles, wind patterns, and rainfall are also influential^18,29,30^, while hormones and other biochemical regulatory mechanisms guide reproduction at the cellular level^31,32^. Additionally, reproduction can be negatively impacted (fewer gametes produced, reduced fertilization, skipping a reproductive season, etc.) by environmental stressors including increasing ocean temperatures and bleaching^12,33–36^, increased sedimentation^9,37^, fresh water inundation^9,38^, and ultraviolet radiation (UVR) exposure^39,40^ among others. The timing of reproduction might even be shifted by changes in environmental conditions^41,42^.

Numerous advances surrounding the field of coral reproduction have been achieved over the last 40 years^7,8,18,19,22,43^. Nonetheless, the basic reproduction of fewer than half of the ~ 1,400 stony coral species around the world is currently well documented^7,19^, and new discoveries are being made while local and global stressors concomitantly impact reproduction^9,10,13,34,44,45^. Successful reproduction and the ensuing larval recruitment are vital for preserving genetic diversity, promoting adaptation and resilience, preventing ecosystem phase shifts, and more^14,46^. These studies are not only crucial to understanding the effects of various stressors on the sexual reproduction and early life history stages of reef populations, they are also vital to informing conservation and management initiatives that may seek to consider reproductive events or metrics as part of an overall program strategy^47–49^. Therefore, in order to understand, protect, and help restore coral reefs around the world, it is essential to know when and how they reproduce, which can be achieved via careful monthly histological examination in conjunction with in situ or ex situ visual observations.

Three genera comprise the majority of Hawaii’s most abundant scleractinian corals: *Porites*, *Montipora*, and *Pocillopora*^50,51^. *Porites compressa* and *Montipora capitata* dominate the coral community in Kāneʻohe Bay, and *M. capitata* is also one of the most abundant species statewide and an important contributor to reef accretion^50–52^. Its congener *Montipora flabellata* is also relatively abundant statewide but typically has a very restricted niche, one of high wave energy, water flow, and high UVR exposure^51,53^. Much is known about the reproduction of a select few, well-studied corals throughout Hawaiʻi and in Kāneʻohe Bay such as *M. capitata*, *Lobactis scutaria*, and *Pocillopora acuta*^26,54,55^, and there is some baseline reproduction information for many other Hawaiian scleractinian coral species^25,56,57^. *Montipora capitata* is known to reproduce on or around nights surrounding the new moon in late May/June, July, and occasionally August where colonies release thousands of egg-sperm bundles in synchronized spawning events^25,54,56,57^. In contrast, little is known about the reproduction of *M. flabellata*, as it has not often been the target of in-depth reproductive analyses^25,56,57^. While *M. capitata* has repeatedly been observed to release thousands of egg-sperm bundles *en masse* over a short period of time, *M. flabellata* that were preliminarily observed throughout July and August 2017 never released more than 40–50 bundles on a given spawning night.

Here we conducted a comparative study of *M. capitata* and *M*. *flabellata* reproduction. Because of the observed differences in the reproductive strategies of these coral species, we were interested in describing the reproductive biology of *M. flabellata* in greater depth and contrasting it to *M. capitata*. To do so, we followed both species’ reproductive cycle with classical histological sampling over a complete year and visually observed their reproductive behavior ex situ in mesocosms over two years. The live, visual observations were conducted nightly from late May through early September in 2018 and June, July, and early August in 2019. Finally, to further understand the diverging reproductive strategies of these corals in situ, reproductive output was estimated for a subset of each species in the bay.

## Materials and methods

### Colony location, selection, collections, and husbandry

**Figure 1:**
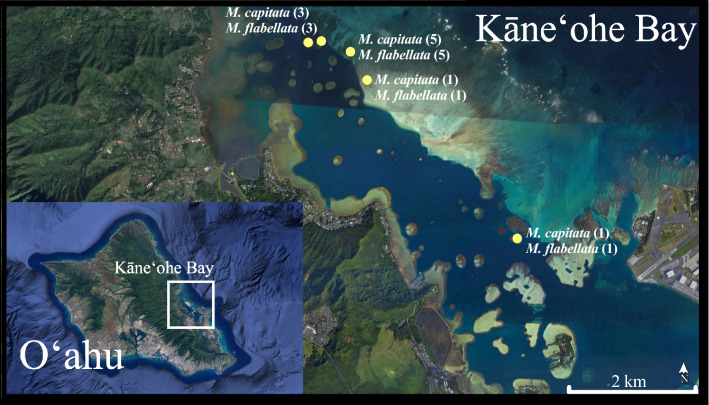
Map of coral collection sites in Kāneʻohe Bay, Oʻahu, Hawaiʻi. Map shows the locations and number of colonies per species used for histological sampling, with each *M*. *flabellata* colony paired to a nearby *M. capitata* colony at the same depth. Additional colonies used for spawning observations were later collected in the same areas. All corals were located at depths between 1 and 4 m. (Google Earth Pro v. 7.3.3.7786, www.earth.google. com).

In October 2017, ten colonies of *M. capitata* (n = 10) and *M. flabellata* (n = 10) were identified, tagged, and GPS marked in locations throughout Kāneʻohe Bay, Hawaiʻi (Fig. 1; 21°47′506″ N, 157°81′914″ W), and these colonies were used in both spawning and histology studies. Because *M*. *flabellata* is less common and more restricted to shallow reef habitat than *M. capitata* in the bay, colonies of both species were selected between 1 and 4 m depth, and each *M*. *flabellata* was paired with a nearby *M. capitata* in an effort to collect from areas of the reef with similar environmental parameters. Coral sample collection was approved by the State of Hawaiʻi Department of Land and Natural Resources under Special Activity Permit Numbers SAP 2018-03, SAP 2019-16, and SAP 2020-25.

### Reproductive behavior and spawning synchrony

To compare the reproductive behavior of the two species, a 20–30 cm section was fragmented from each colony in late May 2018 and again in early June 2019, labeled with its individual number, and maintained in outdoor flow-through seawater tanks at the Hawaiʻi Institute of Marine Biology (HIMB) on Coconut Island in Kāneʻohe Bay. The flow rate of seawater through the mesocosms was approximately 50 L/min, and small powerheads accompanied by a ‘surge tank’ increased water circulation and mimicked the high wave energy environment to prevent sediment accumulating on the colonies. A layer of shade cloth above the tanks provided PAR values ~ 1000–1600 μmol m^−2^ s^−1^ that matched in situ measurements.

Two hours prior to anticipated spawning at 21:00^56,57^, colonies were individually isolated in a seawater-filled container and were returned to their holding tank if there was no release of egg-sperm bundles by 22:30. Spawning observations of *M*. *flabellata* were conducted nightly from late May through early September of 2018, and based on these data, observations were made from June through July in 2019. Spawning of *M. capitata* occurred during new moon phases of those same time periods. The original 10 tagged colonies of each species were tracked over both seasons, but additional colonies were identified and collected to increase sample size for spawning and other reproductive analyses (2018: *M. capitata* n = 10, *M. flabellata* n = 16; 2019: *M. capitata* n = 16, *M. flabellata* n = 14). Because *M*. *flabellata* is sparsely populated throughout the bay and night operations are logistically difficult, it was not possible to also observe spawning in situ.

**Figure 2:**
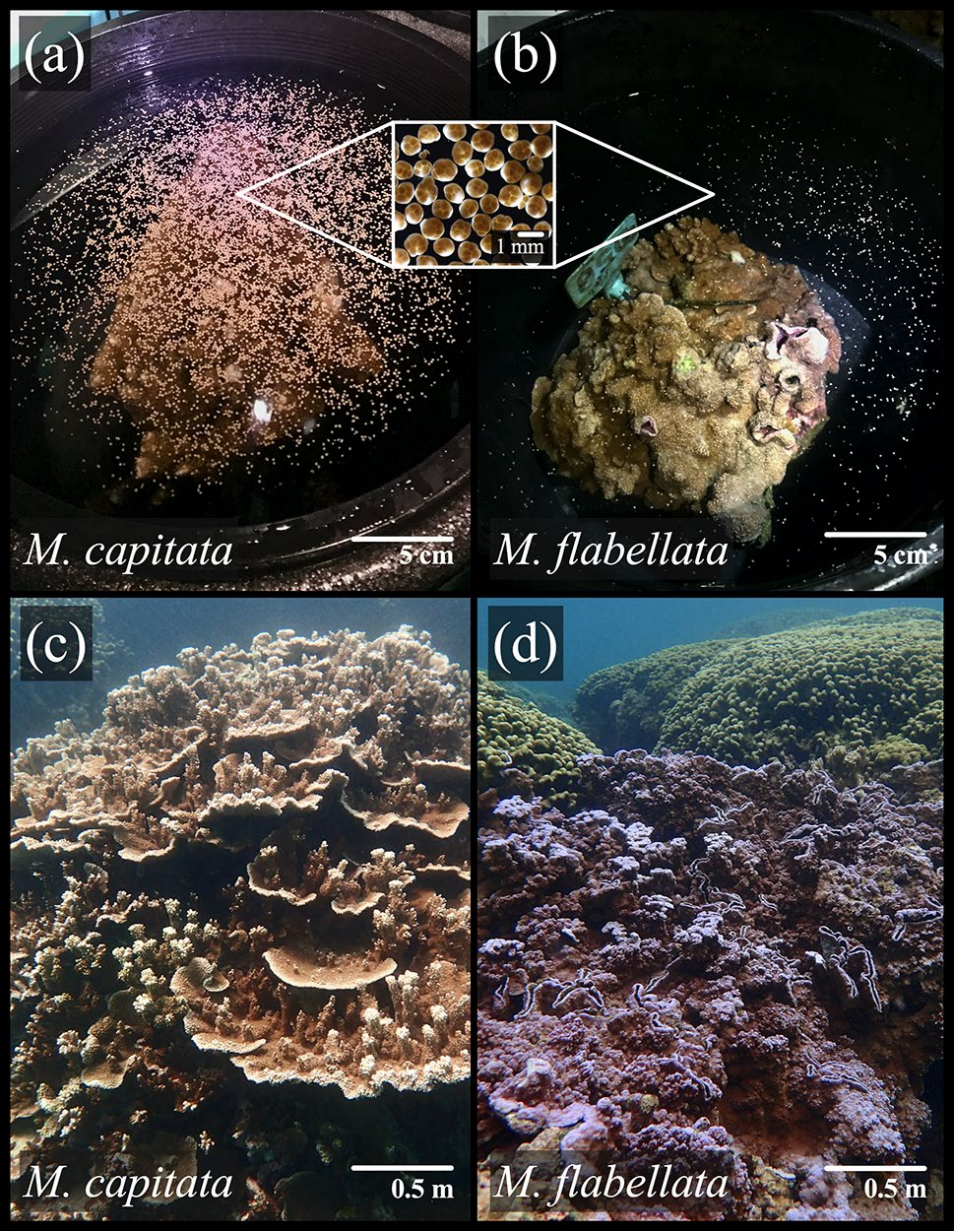
Release of egg-sperm bundles in *M. capitata* and *M. flabellata.* (**a**) Typical spawn from *M. capitata* with egg-sperm bundles covering the surface of the container (approximately 8,000 bundles). (**b**) Example of a “heavy” spawn from *M*. *flabellata* with ~ 400 egg-sperm bundles released. Over two summers, no colonies of *M*. *flabellata* released as many bundles from a heavy spawn in a single night compared to a typical spawn characteristic of *M. capitata*. Inset: close-up of egg-sperm bundles before breaking apart and releasing eggs and sperm to water column. (**c**) *Montipora capitata* and (**d**) *M. flabellata* in Kāneʻohe Bay. All photographs by first author.

While *M. capitata* predictably and reliably releases thousands of egg-sperm bundles *en masse* each spawning season, *M*. *flabellata* colonies from preliminary observations in 2017 released bundles much more sporadically and in far less abundance than expected for broadcast spawners; this atypical broadcast spawning pattern was again observed over the course of this study (2018 and 2019). Therefore, a colony for both species was counted as “having spawned” if a minimum of 10 egg-sperm bundles were released. Individual spawning colonies were later divided into two groups: “light spawning” if it was practical to count the number of bundles released (typically less than ~ 200 egg-sperm bundles) and “heavy spawning” if it was impractical to count the number of bundles released, generally greater than ~ 200 egg-sperm bundles (Fig. 2).

Spawning synchrony was assessed using the Marquis synchrony index adapted for coral reproduction^43,58^. The synchrony index, here calculated at the daily level, considers the proportion of corals spawning on each day that spawning was monitored:

$${S}_{M}=\frac{d1}{\sum_{t=1}^{t=n}dt}\cdot p1+ \frac{d2}{\sum_{t=1}^{t=n}dt}\cdot p2+ \frac{d3}{\sum_{t=1}^{t=n}dt}\cdot p3+\dots + \frac{dn}{\sum_{t=1}^{t=n}dt}\cdot pn$$

where *t* is the day of spawning monitoring, *n* is the number of days monitored, *d*_*t*_ is the number of corals spawning on day *t*, *p*_*t*_ is the proportion of corals that spawned on day *t*, and $\sum_{t=1}^{t=n}dt$ is the total cumulative number of corals that spawned during the period studied. The Marquis index of synchrony ranges from 0 (spawning spread across spawning days with no overlap) to 1 (all corals spawned synchronously on the same days). The synchrony index was primarily based on the nights that involved heavy spawns, integrated with a reduced weight of 16% given to light spawns. The 16% was calculated by dividing the average light spawn bundle number by the average heavy spawn bundle number, representing the average contribution of the light spawns.

Corals were held in mesocosms in conditions designed to minimize stress, support healthy conditions for reproduction, and inspected daily for signs of stress and/or tissue loss. In general, all colonies were maintained in good health with normal coloration and tissue integrity for the entire duration of the study. In the few isolated instances when a lesion appeared, a 2–3 cm area around the lesion was removed, and the area was sealed with two-part epoxy, halting further necrosis to maintain the overall health of the colony. At the conclusion of the study, all corals were returned to their parent colony location and secured to the reef with two-part epoxy.

### Histology

In parallel with the live observations of reproductive behavior, we conducted a year-long series of sample collection and histological examination to examine the growth of eggs and sperm in the tissues of each species. All ten of the originally tagged *M*. *flabellata* colonies (n = 10) were used for histology analysis. Fewer *M. capitata* colonies (n = 3) were ultimately analyzed because the histology of *M. capitata* from Kāneʻohe Bay was well documented by Padilla-Gamiño, et al.^59^. For both species, each colony was at least 50 cm in diameter to ensure they were likely to be of reproductive age and were of sufficient size to accommodate sampling across ten months from November 2017 to October 2018. Each month, three to five, ~ 2 cm samples were fragmented from each tagged colony.

Coral histological samples were collected from the interior portion of a colony (away from the growth margin of the encrusting *M. flabellata*) or deep in the branches (away from the distal end for *M. capitata*) to target polyps that were more likely to be reproductive. Samples were fixed in 4% paraformaldehyde in 0.2 µm filtered seawater and stored at 4 °C for a minimum of 48 h. Samples were decalcified with Cal-Ex™ decalcifier (Thermo Fisher Scientific, Waltham, MA) for 24 h as preparation for embedding in paraffin^60^. Dehydration, embedding, sectioning, and staining of slides was performed by the University of Hawaiʻi Histology and Imaging Core Facility. Samples were dehydrated with xylene alcohols and then embedded in paraffin blocks (Thermo Scientific™ Richard-Allan Scientific™ Histoplast Paraffin, Waltham, MA). Sagittal and cross-section cuts (5–7 μm serial sections) were made with a microtome (Leica RM2235, Leica Biosystems, GmbH) producing ten slides per colony for each month sampled. *Montipora flabellata* slide sections were stained with Masson’s Trichrome, and *M. capitata* samples were processed with hematoxylin and eosin (H&E) stain (Thermo Scientific™ Richard-Allan Scientific™ Gill™ 2 Masson Trichrome and Hematoxylin, Waltham, MA).

Tissues were analyzed microscopically for the presence/absence, size, and developmental stages of oocytes and presence/absence and developmental stages of the testes. Slides were assessed and photographed at 100 × or 200 × magnification using a compound microscope and a computer-aided imaging system (microscope: Olympus BX41, Center Valley, PA; camera: S01-0801A, software: SSView v. × 64, 1.0.5969, Motic Instruments USA, Schertz, TX). Each slide was briefly scanned to determine if oocytes and/or sperm were present in the section. In order to standardize measurements to the widest axis and avoid duplicate sampling, only oocytes sectioned through the nucleolus of the nucleus were photographed, and the section with the largest portion of a particular spermary was assessed. Photographs of oocytes were later analyzed and measured with ImageJ software (version 1.51a, Bethesda, MD, USA) using Feret’s Statistical Diameter and a micrometer set to the appropriate scale^59,61^. Both oocytes and testes were categorized by stage of development (I–IV) using common morphology and size guidelines^59,62–64^. See Table 1 for parameter definitions and Figures S1 and S2 for example stages of oogenesis and spermatogenesis, respectively.

**Table 1:** Histological developmental stage classification for gametogenesis in *M. capitata* and *M. flabellata*^59,62–64^.

Stage	Oogenesis	Spermatogenesis
I	~ 20–70 μm oocyte diameter; round or oval shape nucleus occupies most of oocyte space	Loosely defined clusters of spermatogonia surrounded by mesentary
II	~ 50–300 μm oocyte diameter; vitellogenesis apparent; nucleus still in center of oocyte	Well-defined and more uniform in shape clusters of spermatocytes with larger nuclei
III	~ 200–500 μm oocyte diameter; lipid vesicles easily distinguished; nucleus shifting away from oocyte center	Larger diameter cluster of spermatocytes with prominent central lumen
IV	~ 300–600+ μm oocyte diameter; nucleus at vitelline membrane at periphery of oocyte with nucleolus frequently at periphery of nucleus	Spermatozoa with distinct tails projecting into and occupying lumen space

### Estimates of reproductive output

Because *M*. *flabellata* exhibited an irregular spawning pattern in contrast to *M. capitata*, we sought to understand and compare an estimate of reproductive output on a single night of spawning for each species. This was done by determining: (1) the mean number of bundles released for both “light” and “heavy” spawning for fragments of *M*. *flabellata*, as well as typical for a fragment of *M. capitata* used in this study; (2) the mean size of fragments used for spawning; (3) approximately how many of these average sized fragments would comprise a hypothetical 1 m^2^ adult colony on the reef; and (4) approximately how many bundles could be spawned from a 1 m^2^ adult colony per night for each species.

For *M*. *flabellata*, the mean number of bundles per “light spawn” was determined by counting the total number of bundles released from each colony every night of spawning for both 2018 and 2019 (n = 117 light spawns). For colonies that “heavily spawned” (more than ~ 200 bundles), the total number of bundles was counted or visually estimated for each colony given what remained after collecting ~ 100–200 bundles that were used for other reproductive analyses (n = 44 heavy spawns); the mean number of bundles per heavy spawn was then calculated from those values. The number of bundles released for a characteristic *M. capitata* spawn was estimated by counting a section of a photograph and extrapolating to the whole (Fig. 2).

To estimate spawning coral size, photographs of a subset of corals used for spawning (n = 7) were analyzed with ImageJ software (version 1.51a, Bethesda, MD, USA) to calculate the approximate surface area (cm^2^). This was used to round to an approximation of standard fragment surface area size typically used for spawning. In order to reduce the influence of rugosity on surface area, fragments with a low, sprawling profile and minimal vertical branching growth formation were selected for measurements. Using the mean surface area of spawning fragments, the number of bundles released for each type of spawning (‘light’ vs. ‘heavy’ for *M*. *flabellata* and ‘typical’ for *M. capitata*), and assuming a modestly sized 1 m^2^ adult colony, the reproductive output (in egg-sperm bundles) per night from a single colony was estimated (Table 2).

**Table 2:** Estimated reproductive output (egg-sperm bundles).

Species	Avg frag surface area (cm^2^) ± SD	Approx. bundles/frag, light spawn	Approx. bundles/frag, heavy spawn	Approx. bundles/frag, typical spawn	Est. bundles/colony 1 m^2^ (10,000 cm^2^) surface area
*M. flabellata*	250 ± 70	75	400	–	Light spawn: 3000 Heavy spawn: 16,000
*M. capitata*	250 ± 70	–	–	8000	Typical spawn: 320,000

A comparison of the number of egg-sperm bundles spawned on a given night was estimated for a 1 m^2^ colony of *M*. *flabellata* and *M. capitata*. The mean number of bundles spawned per small colony fragment per night for both years was grouped into light and heavy spawning for *M*. *flabellata* and estimated for a typical *M. capitata* spawn. Given the sporadic spawning behavior of *M*. *flabellata*, similarly sized *M. capitata* colonies typically produce egg-sperm bundles an order of magnitude greater, or more, than *M*. *flabellata* on a given night of spawning.

### Environmental variables

Hourly temperature and light measurements were obtained from the PacIOOS Observing System via the HIMB Weather Station in Kāneʻohe Bay (Rodgers et al., 2005); solar irradiance was measured with a LiCor 200SZ Pyranometer (LI-COR: Lincoln, NE, USA). Due to station outage during the latter portion of the sampling interval (Sep-Oct 2018), temperature and light measurements from 2017 to 2019 were used to calculate a three-year monthly mean value for each parameter.

### Spawning predictor model and statistical analysis

Based on spawning observations and presence of stage IV histology gametes, a main spawning period was identified for each *Montipora* species. For this main spawning period, at least 30% of colonies participated each month. Two environmental predictors were selected for inclusion in a predictive model and calculated at the monthly level: sea surface temperature (SST) and solar irradiance. To determine the spawning predictive capability of each environmental variable, we fitted logistic regression models for each variable. We report the AIC of each model, the Nagelkerke (Cragg and Uhler) pseudo-R^2^, and the model p-value. The analyses were conducted in R^65^, using the packages car^66^ and rcompanion^67^.

Mean oocyte diameter data was square root transformed to meet ANOVA assumptions, normality assumptions visually verified with a Shapiro test, and each species was analyzed with a one-way ANOVA and Tukey’s post hoc analysis. The ANOVAs for mean oocyte diameter per month were analyzed with R^65^, and GraphPad Prism 9 software (version 9.0.1; San Diego, CA) was used to assess colony spawning and gamete stage frequency. All data generated or analyzed during this study are included in this published article and its supplementary information files. All methods were carried out in accordance with relevant guidelines and regulations.

## Results

### Spawning observations and synchrony

**Figure 3:**
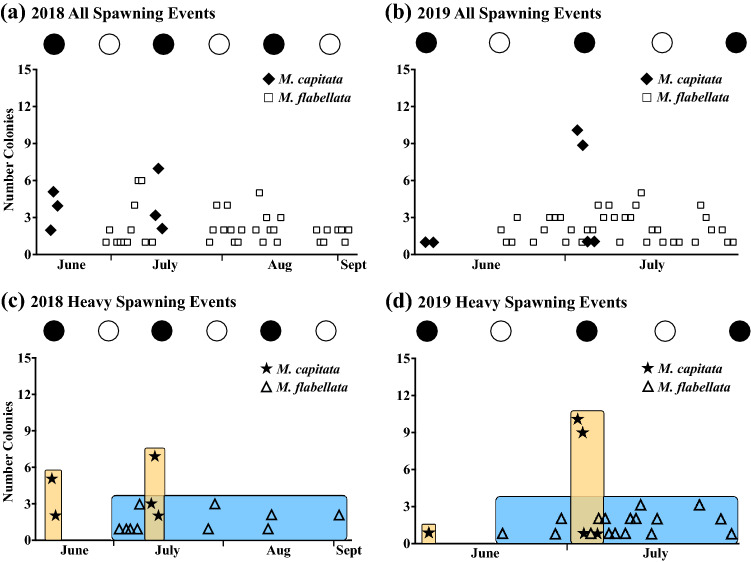
Number of spawning colonies observed per night, 2018 and 2019. All spawning events (light and heavy spawns) with number of colonies spawning per night in (**a**) 2018 and (**b**) 2019 [*M. capitata* (n = 10, 2018; n = 16, 2019) and *M*. *flabellata* (n = 16, 2018; n = 14, 2019)]. (**c**) Total number of colonies per night that only heavily spawned in 2018 and (**d**) 2019. In contrast to *M. capitata*, there were relatively few (at most three) colonies of *M*. *flabellata* that heavily spawned on any given night. Throughout both summers *M. capitata* exhibited large episodic spawning events within a narrow window of a few days in June and July corresponding with the new moon, which is common for the species. By contrast, *M*. *flabellata* showed smaller distributed spawning events with intermittent heavy spawning, low number of colonies spawning per night, and without an apparent lunar cycle cue. Filled circles ﻿ are new moon; empty circles ◯ are full moon.

Both *M. capitata* and *M. flabellata* began spawning at approximately 21:00. In accord with previous observations, *M. capitata* spawned during or a few days after the new moon in the summer months, typically with June and July having the largest pulses of gamete bundles released (Fig. 3). It is common for some *M. capitata* colonies to release a small number of remaining bundles at the new moon in August, consisting of a minor spawning event compared to June and July spawns. However, 2019 was an anomaly as *M. capitata* had only a single large spawning event in July and not two large spawning events per summer that is characteristic of the species (Fig. 3).

It is also common for broadcast spawning corals to release a small number of bundles a day or two before a large, mass spawning event, a behavior often described as “dribbling” and referred to as “lightly spawning” here. Throughout both 2018 and 2019, *M. flabellata* followed this dribbling-type of light spawning behavior whereby colonies released a small number of egg-sperm bundles, almost nightly, over several months (Figs. 2 and 3). In both 2018 and 2019, *M. flabellata* began spawning 1–2 days after the June full moon and continued throughout the summer. In both years, the largest number of *M. flabellata* colonies that spawned bundles on any given night was six (of 16 total) colonies (Fig. 3).

*Montipora flabellata* also never coalesced its reproduction into a large group of heavily spawning colonies akin to *M. capitata* and other simultaneous hermaphroditic, broadcast spawning acroporid corals. At most, three of 16 colonies monitored on a given night were observed to heavily spawn, and if a heavy spawn was observed, it was typically one or two colonies (Fig. 3). Moreover, when *M*. *flabellata* did produce enough bundles to qualify as a “heavy spawn,” the few hundred bundles released were still orders of magnitude fewer in number compared to the thousands of bundles characteristic of *M. capitata* and most other broadcasting corals (Table 2; Fig. 2). Additionally, whereas *M. capitata*’s spawning was linked to the new moon, *M*. *flabellata* did not appear to follow a similarly correlated lunar phase environmental cue. Both light and heavy *M*. *flabellata* spawns were spread throughout most of the summer in both years monitored and without a clearly delineated lunar phase (Fig. 3).

To track individual colonies over time, the total number of light and heavy spawns for the same 10 colonies of *M. capitata* and *M. flabellata* was recorded in 2018 and 2019. In 2018, most *M. capitata* colonies recorded at least one or two heavy spawning nights and five colonies each had one light spawn (Fig. S3a). Because *M. capitata* only experienced one month of spawning in 2019 (instead of the typical two spawns), the total number of spawning events per colony was reduced, but there was still a synchronized heavy spawning event (Fig. S3a). While *M. capitata* spawning behavior among colonies was relatively consistent and somewhat evenly distributed among the observed colonies, two of the ten *M*. *flabellata* colonies accounted for most of the light and heavy spawns both years with high variability across the remaining colonies (Fig. S3b).

The Marquis synchrony index indicated a four-fold lower daily spawning synchrony in *M. flabellata* in comparison with *M. capitata* (Marquis Synchrony Index = 0.12 and 0.49, respectively, 2018 and 2019 averaged together). Spawning synchrony was slightly higher in 2019 than in 2018 in both species, with a 31% percent increase in *M*. *flabellata* (2018 Marquis Synchrony Index: 0.102; 2019 Marquis Synchrony Index: 0.134), and a 11% increase in *M. capitata* (2018 Marquis Synchrony Index: 0.47; 2019 Marquis Synchrony Index: 0.52).

### Histology and gametogenesis

**Figure 4:**
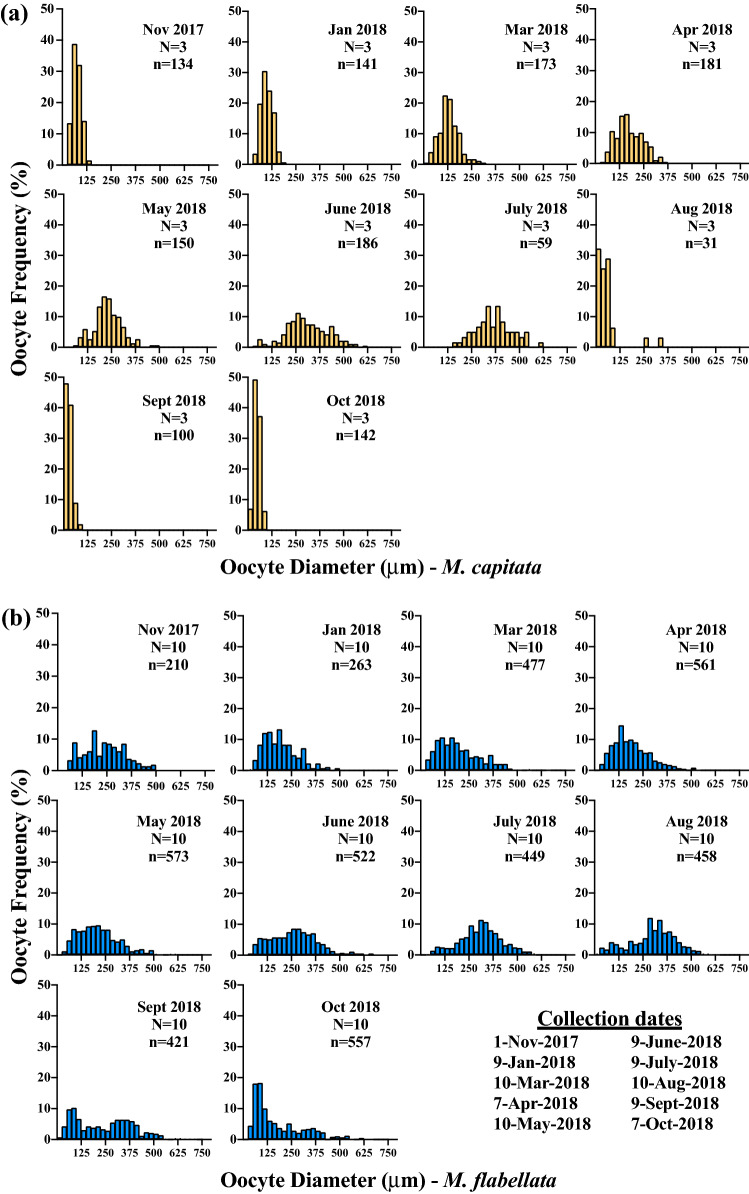
Frequency distribution of oocyte size per month with collection dates. Number of genotypes (N) and number of oocytes (n) analyzed per month for (**a**) *M. capitata* and (**b**) *M. flabellata*. Oocyte diameters are binned into 25 μm increments.

**Figure 5:**
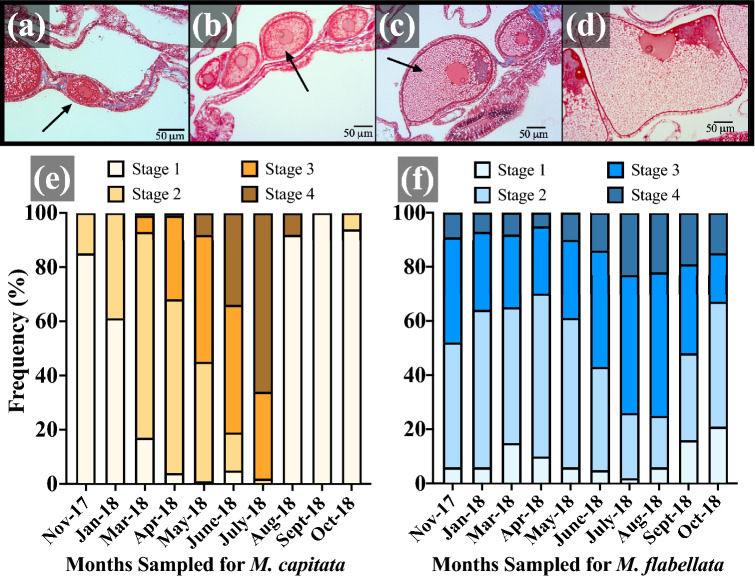
Oocyte developmental stage frequency distribution per month. Stages of oogenesis with nucleus and nucleolus present; arrows point to an oocyte. (**a**) stage I oocyte surrounded by mesoglea; (**b**) stage II oocyte commencing vitellogenesis; (**c**) stage III oocyte with nucleus migrating to periphery; (**d**) stage IV mature oocyte with nucleus at periphery of oocyte and nucleolus at periphery of nucleus; (**e**) *M. capitata* and (**f**) *M*. *flabellata* oocyte stage percent frequency per month sampled. All scale bars 50 μm.

Oocyte maturity in *M. capitata* culminated with the highest frequency of mature oocytes in June/July in *M. capitata* and July/August in *M. flabellata* (Figs. 4 and 5). Oogenesis occurred over a period of approximately 10 months beginning late summer/early fall, and the summer months of June to August also had the largest variation in oocyte size due to mature Stage IV oocytes (~ 300–600+ μm diameter) occurring alongside early Stage I (~ 20–70 μm) oocytes (Table 1; Figs. 4a and 5a–e). *Montipora flabellata* had the highest frequency of mature oocytes in July and August, but the variation in oocyte size extended over a much longer period; large, mature oocytes were found throughout the late fall, winter, and early spring months (November to March), though in lower frequency than the summer months (Figs. 4b and 5f). Although there was a mid to late summer peak in oocyte diameter, there was not a dramatic decline in either oocyte size or stage frequency that marked the end of a reproductive season for *M. flabellata*, as seen in *M. capitata* beginning in August (Fig. 4 and 5).

**Figure 6:**
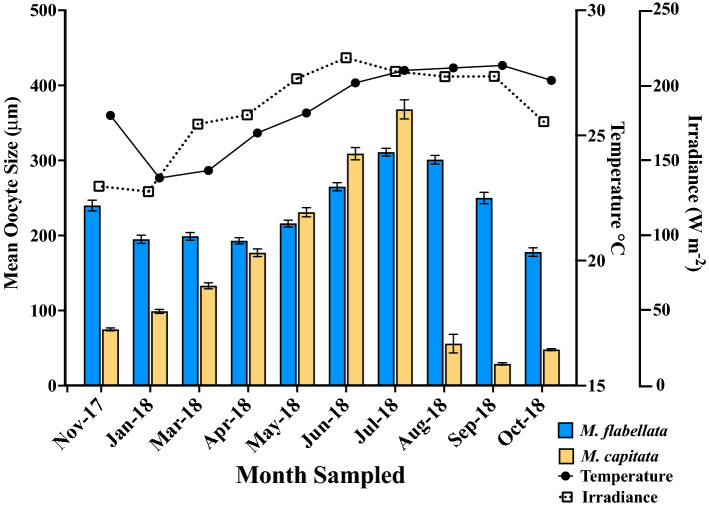
Oogenesis, temperature, and solar radiation. Comparison of mean oocyte size per month with monthly temperature and irradiance means, *M. capitata* (n = 3 genotypes per month) and *M*. *flabellata* (n = 10 genotypes per month). Error bars are ± SEM.

A one-way ANOVA with Tukey’s post hoc comparison of mean oocyte diameter by month determined that oocyte size was largest in June and July for *M. capitata* (Figs. 6 & S4a; F_9,1287_ = 430.9, *p* < 0.0001) and in July and August for *M*. *flabellata* (Figs. 6 and S4b; F_9,4481_ = 73.94, *p* < 0.0001). Additionally, there was a noticeable increase in oocyte diameter per month in *M. capitata* that began in November and continued through spawning in July, with a sharp decline in size and presence of mature oocytes after spawning (Figs. 6 & S4a). In contrast, while *M. flabellata* mean oocyte size did decline beginning in October, a similar dramatic decline and absence of mature oocytes was not observed, further indicating that *M*. *flabellata* had a cohort of oocytes spanning various stages of immature to mature development throughout the year (Fig. 6 and S4b).

**Figure 7:**
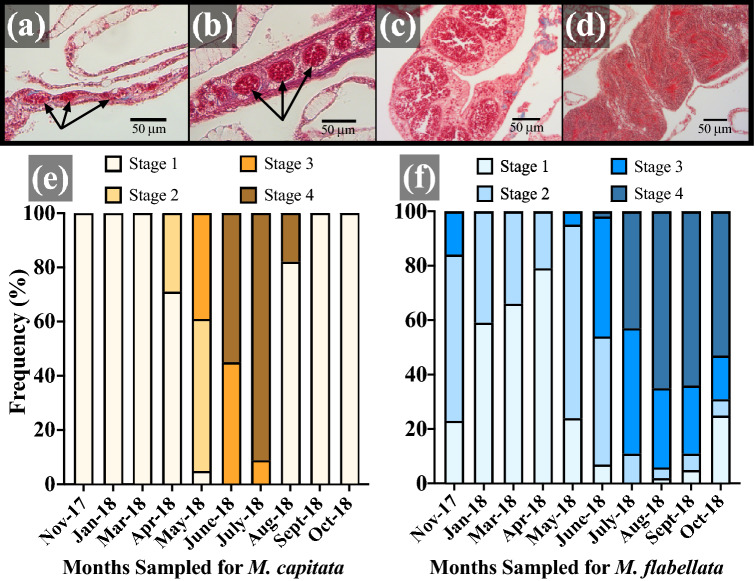
Sperm developmental stage frequency distribution per month. Stages of spermatogenesis; arrows point to a cluster of developing male germ cells. **(a)** stage I spermaries with cluster of spermatogonia; (**b**) stage II spermaries with spermatocytes; (**c**) stage III spermaries with spermatocytes around central lumen; (**d**) stage IV spermaries with spermatozoa tails projecting into lumen; (**e**) *M*. *capitata* and (**f**) *M*. *flabellata* spermary stage percent frequency per month sampled. All scale bars 50 μm.

Spermatogenesis (Table 1; Fig. 7a–d) in *M. capitata* began in April and continued through midsummer. Mirroring oocyte development, mature sperm occurred in their highest frequency in June and July, and a small portion of Stage IV spermatocytes remained in August, corresponding to the minimal amount of spawning typically observed near the end of summer; noticeable testes development were absent from September through March (Fig. 7e). As with oocytes in *M*. *flabellata*, there was also an expanded window for spermatogenesis with mature sperm appearing in June and found in highest proportion from July through October (Fig. 7f). There was a noticeable reduction in Stage III and IV spermatocytes beginning in November, and only immature Stage I and II spermaries were present from January to April (Fig. 7f). Unlike *M. capitata*, spermaries did not disappear from *M. flabellata* and instead were found in various stages of development throughout the year.

### Estimated reproductive output

Table 2 summarizes the estimates of comparative reproductive output, in terms of number of egg-sperm bundles, for *M*. *flabellata* and *M. capitata*. Mean fragment size surface area used for spawning for both species was ~ 250 cm^2^. The combined 2018 and 2019 mean number of bundles produced by *M. flabellata* was 64 bundles per colony for a light spawn and 395 bundles per colony for a heavy spawn, rounded to 75 bundles and 400 bundles, respectively. Approximately 8,000 bundles were released during a ‘typical’ spawn characteristic of a similarly sized *M. capitata* fragment. Assuming an adult *M. flabellata* colony with a 1 m^2^ surface area, an estimated range of 3000 bundles (‘light’ spawn) to 16,000 bundles (‘heavy’ spawn) could be expected to be produced. By contrast, a similarly sized *M. capitata* colony would release ~ 320,000 bundles on a given night of spawning characteristic of the species, representing an estimated reproductive output that is 20 times greater than a heavy *M. flabellata* spawn.

### Environmental predictors for spawning

Our observations of spawning and presence of stage IV gametes allowed us to identify the main spawning period for *M. capitata* (June and July) versus *M. flabellata* (July to October). Two environmental variables, SST and irradiance, were tested as potential predictors of spawning for each *Montipora* species in this study. In *M. capitata*, irradiance best predicted whether corals spawned each month (AIC = 4.00, pseudo R^2^ = 1.00, *p* = 0.001) whereas in *M*. *flabellata*, SST was the best predictor for whether corals spawned (AIC = 4.00, pseudo R^2^ = 1.00, *p* < 0.0001). See Tables S1–S3 and Figure S5.

## Discussion

Both *M. capitata* and *M. flabellata* are simultaneous hermaphrodites that broadcast spawn egg-sperm bundles. Padilla-Gamiño, et al.^59^ originally described the annual gametogenic cycle of *M. capitata* via histological analysis, and our results corroborate their previous findings. The oocytes and testes of *M. capitata* matured and displayed a precipitous decline in size and number commensurate with the brief window of summer spawning observations cued to the new moon. Padilla-Gamiño, et al.^59^ attributed *M. capitata*’s periodic monthly split spawning and variation in gamete maturity within a colony to both partial colony spawning and intra-colony variability. They found that irradiance, not temperature, had the strongest correlation to oocyte size. Despite the 2014 and 2015 consecutive warming events that caused widespread bleaching in Kāneʻohe Bay^68,69^, we found no appreciable shift in spawning or gametogenesis since their initial reproductive analyses of *M. capitata* nearly a decade ago.

In contrast, there was no clearly defined beginning or end of gametogenesis in *M*. *flabellata*. Temperature was the best seasonal predictor of spawning in *M. flabellata*. We also discovered that gamete development among and within colonies was characterized by even greater variability across an even broader gametogenic cycle than *M. capitata*. *Montipora flabellata* had a diverse size and maturity range of oocytes yearlong with an increase in proportion of mature oocytes during the midsummer that gradually declined into the early fall months, but large mature oocytes were always present, unlike *M. capitata*. Testes in *M. flabellata* matured in conjunction with oocytes and were also observed in varying stages throughout the year, but, unlike oocytes, there were no late-stage spermatocytes in the winter and spring.

*Montipora flabellata* spawning was spread sporadically throughout much of the summer and did not seem to strongly correlate with a particular moon phase. Additionally, there was never a coordinated whole-colony release of bundles *en masse* involving numerous individuals on a single night or few nights in succession that is characteristic of *M. capitata* and other acroporids^8,18,19,22,54^. Typically, small clusters of bundles in varying amounts, often from only one or a few colonies, were released intermittently from late June through September, yielding reproductive output orders of magnitude lower and reproductive synchrony levels less than a quarter than that of *M. capitata*. While mature oocytes were still present, the absence of mature sperm in the winter months suggests a possible cessation of spawning activity after October or early November, but continual visual observations throughout the fall and winter months are needed to confirm this.

To determine what time of year a species of coral is most likely to spawn, the focus of gamete maturation for simultaneous hermaphrodites is typically channeled into analysis of oocyte presence and development^70–73^. This is understandable since large, lipid-fueled oocytes are energetically expensive compared to relatively metabolically inexpensive sperm production^74,75^. Additionally, simple and low-cost methods can be utilized for coral species whose pigmented, mature oocytes are visible to the naked eye or easily examined with a low power dissecting microscope. Branches or small sections of *Acropora* and some *Montipora* species can be quickly examined for presence/absence of mature eggs^71^ whereas the costly time and financial investment required to histologically examine the stages of sperm maturation can be prohibitive. With sperm and oocyte maturation closely linked, only primordial and early-stage oocytes were present after the spawning season in *M. capitata*, and noticeable testes development did not appear until the following spring (Figs. 5 and 7; Padilla-Gamiño, et al.^59^). For *M. capitata*, as with so many other acroporids, we have observed that visual presence of pink-pigmented oocytes is a reliable indicator of colony maturity and reproductive readiness. Within *M*. *flabellata* on the other hand, mid and late stage oocytes were found all year in relatively high abundance, but there was a clear absence of late stage spermaries from winter until maturation in early summer (Figs. 5 and 7). Interestingly, however, even though late stage, mature spermaries disappeared from *M*. *flabellata* in the winter months, easily identifiable mid stage spermaries were found year-round as well (Fig. 7). Perhaps, then, high abundance of mature sperm—not simply presence/absence of mature oocytes—in *M. flabellata* is a better predictor of spawning season.

It seems anomalous that *M*. *flabellata* colonies contain mature eggs in winter with no mature sperm; however, its niche habitat, colony morphology, and skeletal matrix might provide some context. While *M. capitata* can be found throughout a range of reef habitats with associated morphological plasticity, *M*. *flabellata* has an encrusting growth form, occasionally forming plates along the margins, and is primarily restricted to shallow areas subject to high light and high wave energy^53,76^. Richards Donà^53^ described its thin, porous skeleton as similarly found in other competitive species characterized by high growth rates and susceptible to fragmentation, yet also highly sensitive to bleaching and mortality^77^. Since mature sperm are absent from late fall through early summer, oocytes found in various stages of development might serve as lipid storage during the winter months when storm and wave surge are highest and colony breakage is most likely to occur. Okubo, et al.^78^ observed reabsorption of oocytes across varying sizes of fragments in sexually mature colonies of *Acropora formosa*; early-stage oocytes were more likely to be reabsorbed than late stage, indicating a possible energy allocation trade-off between survival of the fragment and reproduction. Reabsorption of oocytes has been reported in other corals as well^79–81^. Hawaii’s shores are famous for their winter ‘big wave’ surf in habitat characteristic of *M*. *flabellata*. Perhaps a range of oocyte sizes both within a polyp and throughout a colony can serve as energy stores for reattachment, repair, and regrowth that correlates with an increased probability of fragmentation during a time of year when wave energy is highest. Additionally, if a large fragment is split from the main colony during the winter storm season, a mixed cohort of developing oocytes might even still allow for that fragment’s investment in sexual reproduction the following reproductive season while simultaneously serving as energy for reattachment and growth.

In general, large, synchronized pulses of gamete release are expected to increase the chance of successful fertilization for benthic invertebrates^20,21,82–84^. For corals, the timing of spawning within each month is often associated with the lunar cycle^18,26,27^ supported by cryptochrome photoreceptors in corals that detect blue light from the moon^85^. With no discernable connection to a lunar phase and no clear peak spawning window, examination of the photoreceptors of *M*. *flabellata* might illuminate the extent of their capacity to detect moonlight and the degree of its influence on reproductive behavior. Its spawning behavior might also be more closely tied to a combination of other environmental variables such as winds, current speed, or tidal changes^18,27,29,30,86^, rather than moon cycles. We show here, for example, that spawning in *M*. *flabellata* was best predicted by sea surface temperatures, which remained at 25.6 °C and above during its spawning period. However, further work on environmental predictors is still needed to include additional environmental variables and to assess the temporal variability of the predictions.

Even though it is an acroporid, *M. flabellata*’s distributed gametogenic cycle, low spawning synchrony, and apparent lack of adherence to a lunar phase spawning cue is atypical compared to most other acroporids and conventional broadcast spawners. Extended reproductive seasons have been described from Kenyan reefs^72^ and on Lizard Island, Great Barrier Reef, where the spawning season of *Acropora* assemblages can last five months or more^72,87^. Additionally, both a fungiid coral from the Galápagos^88^ and a Caribbean reef sponge^89^ were also reported to have extended periods of reproductive activity and spawning of gametes that were not reliably adjusted to the lunar cycle. Unlike *M*. *flabellata*, however, they are both gonochoric, and fertilization for the sponge specifically could be external or internal^89^, an option not available for hermaphroditic broadcasting corals. Chamberland, et al.^90^ reported the Caribbean broadcasting coral *Diploria labyrinthiformis* to spawn for six consecutive months, but that species still had an identifiable, narrow four-day spawning window 10–13 days after the full moon each month. Additionally, unlike some corals that can asexually produce larvae^91,92^, *M*. *flabellata* does not self-fertilize^93^, and there were no observations of developing planulae in the histological sections.

Successful fertilization for broadcast spawners, like *M*. *flabellata*, is influenced by population density, size, and proximity of reproductively mature colonies, as well as gamete numbers, density, rate of dilution, and other physiological and environmental factors^20,83,94^. Large numbers of gametes not only increase the likelihood of fertilization but also minimize the impact of predation^95^. Given the irregular spawning of *M*. *flabellata* colonies over several months, it seems that the goal of successful fertilization is already impeded by low synchrony, but it is even further complicated by low numbers of egg-sperm bundles released when an individual colony does spawn on any night. A small volume of egg-sperm bundles produced from an aperiodic spawning population would not appear to be a viable reproductive strategy. However, perhaps the stochastic environment characteristic of its shallow water, high wave energy habitat has driven a variable spawning behavior to coincide when local conditions are favorable to reproduction. The resulting extended reproductive readiness or batch spawning may serve as a mechanism to protect investments in reproduction similarly found in other tropical species^96,97^. Even if this is the case, because they do not self-fertilize^93^ colonies would need to be of sufficient size and density to prevent gamete dilution and yield successful fertilization.

Fast growing and highly competitive, some colonies of *M*. *flabellata* have been recorded to reach 2.5–3 m^2^^53^. A 250 cm^2^ fragment from a large colony could release ~ 75–400 bundles per night. At that size and scale of spawn, a 2.5 m^2^ colony could, by estimation, partially spawn 7500–40,000 bundles per night, and 50 similarly sized large colonies occupying a small stretch of reef could release an estimated 375,000–2 million bundles per night over dozens of midsummer night’s spawns spanning several months (Table 2; Figs. 2 and 3). From the 2014 surveys of about half of the patch reefs in Kāneʻohe Bay, Richards Donà^53^ recorded a total of 359 M. *flabellata* colonies on 22 patch reefs inside the bay. Of those colonies, 112 (~ 30%) were in a size class likely to be considered reproductively mature, and two, relatively small, close patch reefs had the highest number (39 and 49) of colonies of reproductive size^53^. Perhaps historically this competitive species existed in sufficiently large colony sizes and numbers that its sporadic reproductive behavior could still yield ample larvae and recruits. The﻿﻿ colonies found inside the bay probably represent the latter end of suitable habitat and population distribution, and this species has previously been more abundant on the outer barrier reef^53,98^. Specialized to a particular habitat and prior to anthropogenic disturbance, *M*. *flabellata* may have been able to occupy vast areas of relatively narrow bands of reef. However, this high wave energy environment could be detrimental to reproduction if, for example, spawning occurred on a night of high current velocity or surface winds which could disperse gamete bundles too quickly^20^. If *M*. *flabellata* was growing in adequate colony size and density, then multiple small spawning events might lead to sufficient successful reproduction instead of relying on a few nights each year where reproductive failure could be high in a dynamic environment.

For benthic, sessile marine organisms, sexual reproductive synchrony is one of the mechanisms that increases the likelihood of successful fertilization, supplies the reef with the next generation of recruits, and is crucial for adaptation^20,21,82–84^. Synchronized and multispecies spawning events have become a hallmark of broadcast spawning corals. *Montipora capitata* reliably fulfills this expectation whereas *M*. *flabellata* seems to be anomalous. As alluded to earlier, this unconventional method of reproduction might have been sufficient—even advantageous—in its high energy habitat prior to widespread anthropogenic impacts. However, its fast, competitive growth appears to be offset by its increased sensitivity to bleaching events, often leading to high partial or whole colony mortality^53,99^. A decline in population density could produce low gamete concentrations resulting in reproductive failure via an Allee effect^100,101^. Extended periods of recruitment failure could put this species at risk, justifying the need for intervention strategies, targeted propagation, or increased monitoring efforts. Kāneʻohe Bay already has a long history of human impact^4^, and the oceans are predicted to continue warming, making bleaching more frequent, severe, and leaving less recovery time between events^102^. If populations of *M*. *flabellata* become fragmented with isolated colonies, its reproductive strategy could feasibly become a liability in the Anthropocene.

## Supplementary Information

Supplementary Information 1.

Supplementary Information 2.
