# Supplementary material for: Contrasting reproductive strategies of two Hawaiian Montipora corals
Source: Sci Rep. 2022 Jul 18;12:12255. doi: 10.1038/s41598-022-16032-6 (PMC9293913; doi:10.1038/s41598-022-16032-6)
Supplement: Supplementary file 1 — Supplementary Information 1. [file 41598_2022_16032_MOESM1_ESM.pdf]

Supplementary Information

Contrasting Reproductive Strategies of two Hawaiian *Montipora* Corals

E.M. Henley, M. Quinn, J. Bouwmeester, J. Daly, C. Lager, N. Zuchowicz, D.W. Bailey, and M. Hagedorn

Send correspondence to: henleym@si.edu or emhenley@hawaii.edu

This file includes:
Tables S1 to S3: results of logistic regression on environmental predictors for spawning and binomial spawning results for *Montipora capitata* and *Montipora flabellata*.

Figures S1 to S5: gamete stage identification for *M. capitata* and *M. flabellata*, number of nights spawning observed for the same 10 genotypes for two years (2018 and 2019), ANOVA comparison of monthly mean oocyte size, and environmental predictors for spawning for both species.

Additional supplementary information for this manuscript:
Excel data file, Supplementary Data, for *M. capitata* and *M. flabellata*.

- Number of colonies spawning per night, 2018 and 2019
- Number of spawning per individually tagged colony, 2018 and 2019
- Histology measurement data for Feret Diameter of oocyte size per month
- Percent of gametes per developmental stage per month
- Reproductive output (egg-sperm bundles) estimate
- Monthly temperature (°C) and solar irradiance (W m^{-2}), 2017-2019

Table S1: Results of logistic regression model on environmental predictors for spawning.

	AIC	Pseudo R^2	Model p-value	
M. capitata				
SST (°C)	12.03	0.35	0.095	
Irradiance (W m^{-2})	4.00	1.00	0.0010	**
M. flabellata				
SST (°C)	4.00	1.00	9×10^{-5}	***
Irradiance (W m^{-2})	15.53	0.37	0.053	

Table S2: Monthly environmental data and binomial spawning outcome, *M. capitata*.

Month	SST (°C)	Irradiance (W m^{-2})	Spawn
Nov-17	25.8	132.3	0
Dec-17	23.9	121.5	0
Jan-18	23.3	128.6	0
Feb-18	23	119.3	0
Mar-18	23.6	173.7	0
Apr-18	25.1	179.7	0
May-18	25.9	203.8	0
June-18	27.1	217.8	1
July-18	27.6	208.7	1
Aug-18	27.8	205.3	0
Sept-18	27.8	205.4	0
Oct-18	27.2	175.3	0

Table S3: Monthly environmental data and binomial spawning outcome, *M. flabellata*.

Month	SST (°C)	Irradiance (W m^{-2})	Spawn
Nov-17	25.8	132.3	0
Dec-17	23.9	121.5	0
Jan-18	23.3	128.6	0
Feb-18	23	119.3	0
Mar-18	23.6	173.7	0
Apr-18	25.1	179.7	0
May-18	25.9	203.8	0
June-18	27.1	217.8	0
July-18	27.6	208.7	1
Aug-18	27.8	205.3	1
Sept-18	27.8	205.4	1
Oct-18	27.2	175.3	1

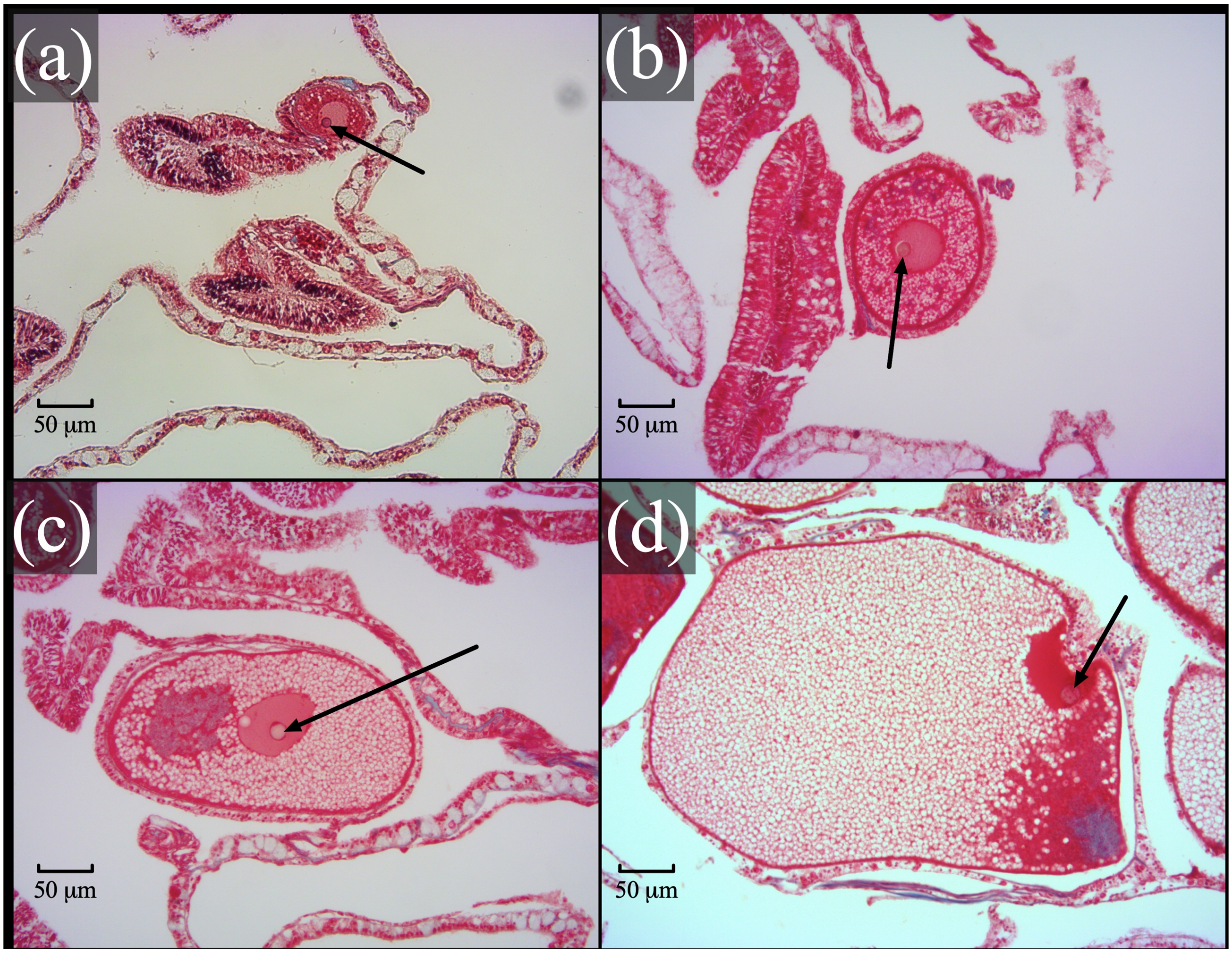

Figure S1. Stages of oogenesis with nucleus and nucleolus present, *M. capitata* and *M. flabellata*. (a) stage I, immature oocyte; **(b)** stage II, oocyte beginning vitellogenesis; **(c)** stage III, oocyte with large lipid vesicles; **(d)** stage IV, mature oocyte with nucleus and nucleolus at periphery. Arrows point to the nucleolus. All scale bars 50 µm.

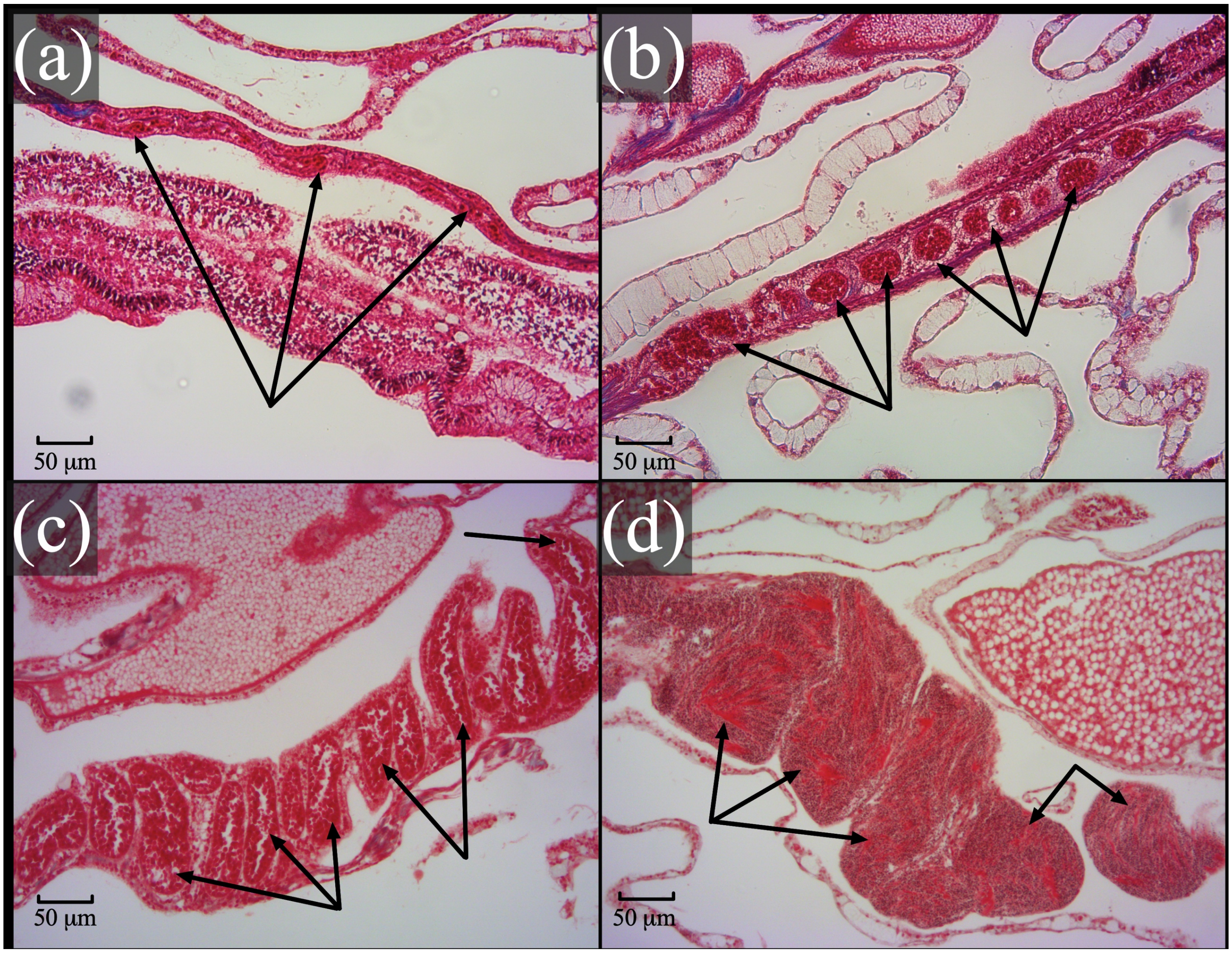

Figure S2. Stages of spermatogenesis, *M. capitata* and *M. flabellata*. (a) stage I, immature spermary; **(b)** stage II, clusters of developing spermatocytes become more well defined; **(c)** stage III, central lumen of each cluster becomes more defined; **(d)** stage IV, mature spermary with tails of spermatozoa filling the lumen. Arrows point to the developing spermary. All scale bars 50 µm.

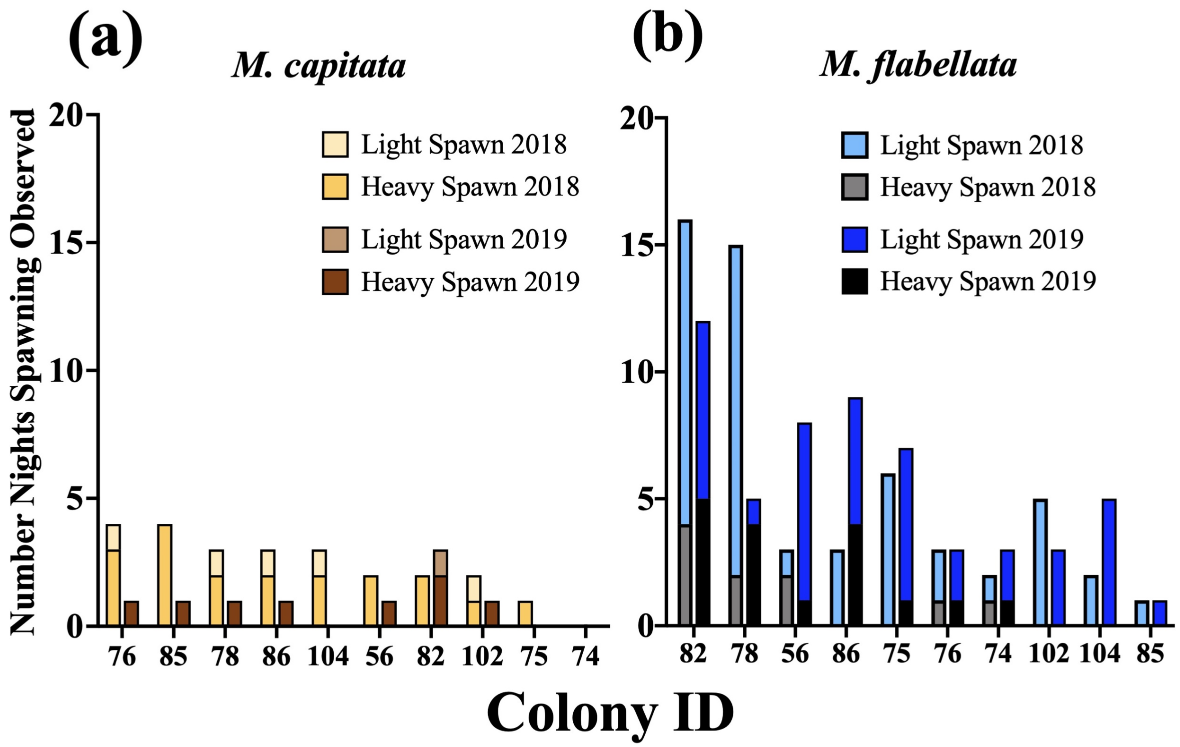

Figure S3. Number of nights spawning observed per colony, 2018 and 2019. The same 10 colonies used for histology sampling were also tracked for spawning and reproductive output over two years. **(a)** *M. capitata* (n = 10 genotypes) and **(b)** *M. flabellata* (n = 10 genotypes). *Montipora capitata* colonies have more consistent spawning behavior whereas *M. flabellata* spawning is much more variable per individual colony.

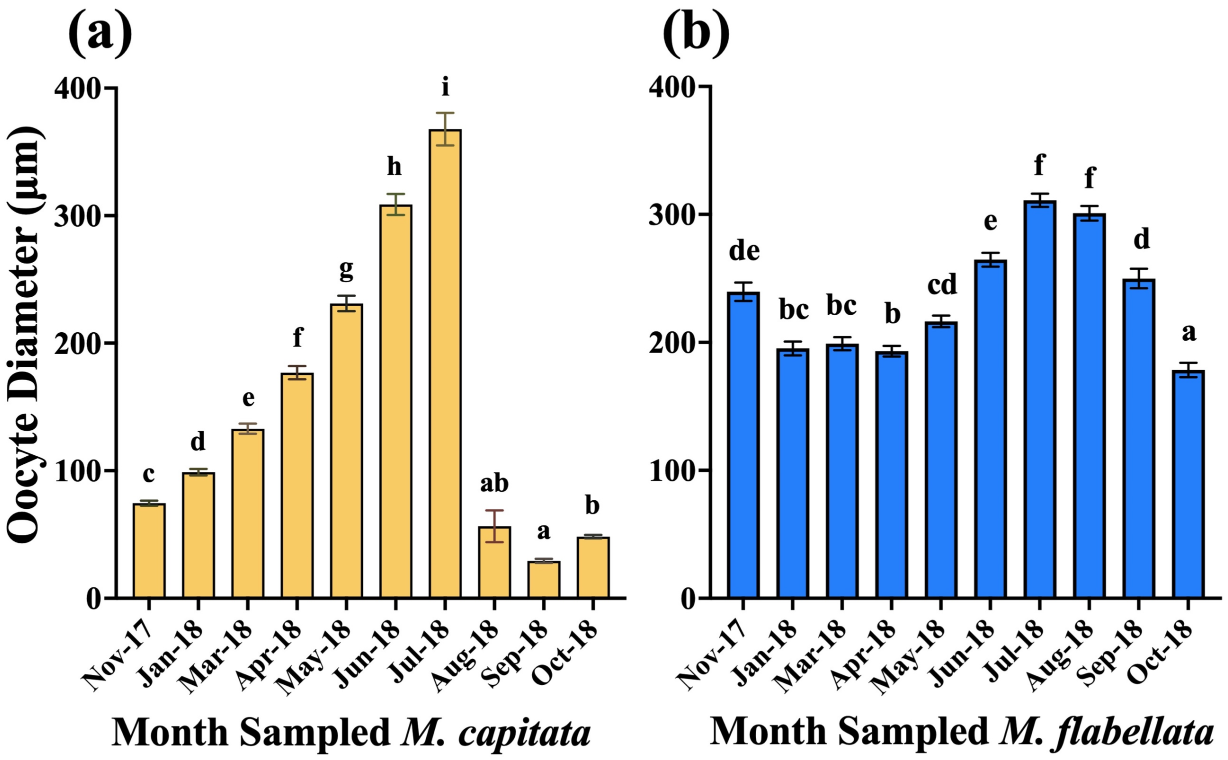

Figure S4. ANOVA of mean oocyte size per species per month.
(a) *M. capitata* (n = 3 colonies per month) one-way ANOVA with Tukey‘s post hoc analysis ($F_{9,1287}$ = 430.9, $p < 0.0001$). The number of oocytes analyzed per month ranged from 31 to 186. **(b)** *M. flabellata* (n = 10 colonies per month) one-way ANOVA with Tukey’s post hoc analysis ($F_{9,4481}$ = 73.94, $p < 0.0001$). The number of oocytes analyzed per month ranged from 201 to 573. The change in size of oocytes per month was more pronounced in *M. capitata* than *M. flabellata*. *Montipora capitata* had a sharp decline in oocyte size from July to August, indicating an end to the spawning season, whereas *M. flabellata* did not have a similar decline. Error bars are ± SEM, and different letters represent differences in statistical significance.

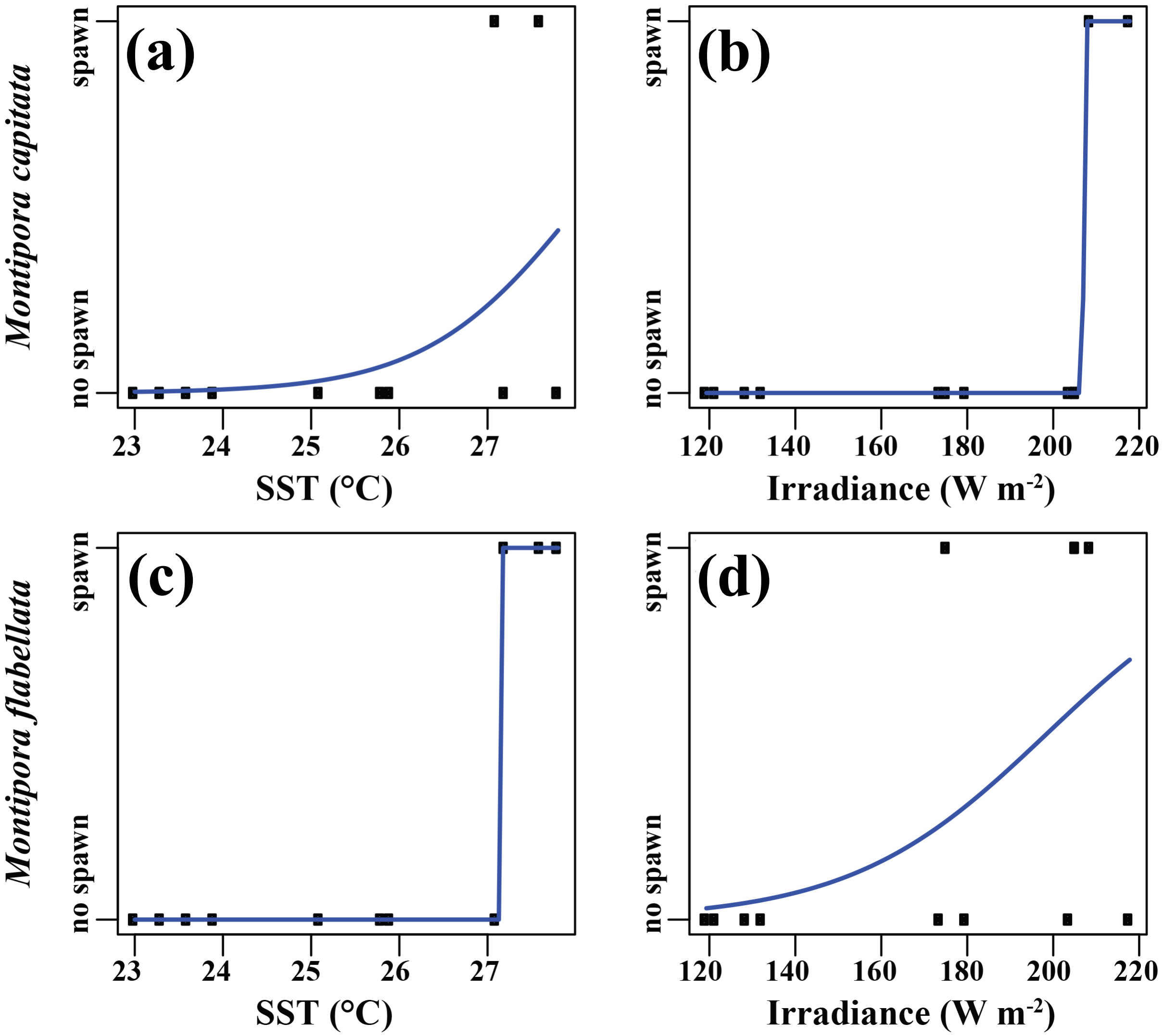

Figure S5. Significant models for spawning predictors for each *Montipora* species. **(a)** Sea surface temperature as predictor for *M. capitata* spawns; **(b)** Solar irradiance as predictor for *M. capitata* spawns; **(c)** Sea surface temperature as predictor for *M. flabellata* spawns; **(d)** Solar irradiance as predictor for *M. flabellata* spawns. Irradiance is a better predictor of spawning for *M. capitata* whereas temperature is a better predictor for *M. flabellata*.
